# Comparing in-person and webinar delivery of an immunization quality improvement program: a process evaluation of the adolescent AFIX trial

**DOI:** 10.1186/1748-5908-9-21

**Published:** 2014-02-18

**Authors:** Melissa B Gilkey, Jennifer L Moss, Alyssa J Roberts, Amanda M Dayton, Amy H Grimshaw, Noel T Brewer

**Affiliations:** 1 Lineberger Comprehensive Cancer Center, University of North Carolina, CB 7440, Chapel Hill, NC 27599, USA; 2 Department of Health Behavior, Gillings School of Global Public Health, University of North Carolina, CB 7440, Chapel Hill, NC 27599, USA; 3 Immunization Branch, North Carolina Division of Public Health, 5601 Six Forks Rd, Raleigh, NC 27609, USA

**Keywords:** Adolescent health services, Immunization programs, Health care quality, access, and evaluation, Quality improvement, Process assessment

## Abstract

**Background:**

Immunization quality improvement programs are often limited by the cost and inconvenience associated with delivering face-to-face consultations to primary care providers. To investigate a more efficient mode of intervention delivery, we conducted a process evaluation that compared in-person consultations to those delivered via interactive webinar.

**Methods:**

The Centers for Disease Control and Prevention’s Assessment, Feedback, Incentives, and eXchange (AFIX) Program is an immunization quality improvement program implemented in all 50 states. In 2011, we randomly assigned 61 high-volume primary care clinics in North Carolina to receive an in-person or webinar AFIX consultation focused on adolescent immunization. We used surveys of participating vaccine providers and expense tracking logs to evaluate delivery modes on participation, satisfaction, and cost. Clinics served 71,874 patients, ages 11 to 18.

**Results:**

Clinics that received in-person and webinar consultations reported similar levels of participation on key programmatic activities with one exception: more webinar clinics reported improving documentation of previously administered, ‘historical’ vaccine doses. Both in-person and webinar clinics showed sustained improvement in confidence to use reminder/recall systems (both *p* < 0.05). Participants rated delivery modes equally highly on satisfaction measures such as convenience (mean = 4.6 of 5.0). Delivery cost per clinic was $152 for in-person consultations versus $100 for webinar consultations.

**Conclusions:**

In-person and webinar delivery modes were both well received, but webinar AFIX consultations cost substantially less. Interactive webinar delivery shows promise for considerably extending the reach of immunization quality improvement programs.

**Trial registration:**

Clinicaltrials.gov, NCT01544764

## Background

Adolescent immunization has emerged as a focus of healthcare quality improvement for several reasons. Adolescents in the U.S. are under-immunized. Despite national guidelines for routine administration, coverage for vaccines in the adolescent platform ranges from 85% for tetanus, diphtheria, and pertussis (Tdap) to 74% for meningococcal conjugate to just 33% for completion of the three-dose human papillomavirus (HPV) vaccine series (females only) [1]. These coverage levels are dramatically lower than for childhood vaccines, which typically achieve coverage over 90% [2]. To improve levels of adolescent vaccination, clinic-level interventions may be particularly effective, since underuse appears to stem, in part, from organizational and provider factors. For example, a leading reason that adolescents are not up-to-date is that providers do not recommend adolescent vaccines in a timely manner [1,3-5].

The recent widespread adoption of immunization information systems, or vaccine registries, has provided a means to address underuse [6]. These secure, electronic databases offer access to population-based coverage data needed for clinic-level benchmarking and evaluation. Registries also support patient reminder/recall systems and other strategies for improving vaccine delivery. Thus, a persistent problem and the increasing availability of tools to address that problem combine to make adolescent immunization a compelling target for quality improvement.

One model used to guide immunization quality improvement for adolescents is the Centers for Disease Control and Prevention’s (CDC) Assessment, Feedback, Incentives, and eXchange (AFIX) Program [7]. Developed to improve immunization in early childhood, AFIX employs an ‘assessment and feedback’ approach that involves evaluating clinics’ vaccine coverage levels and then delivering brief quality improvement consultations to vaccine providers so as to address clinic-specific challenges to immunization. Based on evidence that AFIX raises early childhood immunization coverage by four to seven percentage points above secular trends [8-10], CDC recommends that state and regional health departments provide AFIX consultations to at least one-quarter of federally-funded vaccine providers each year. To maximize reach, larger clinics are prioritized, and many health departments succeed in not only meeting, but greatly exceeding CDC’s 25% goal. More recently, health departments have begun using a modified version of AFIX in an attempt to address the underuse of adolescent vaccines. Research to support this work is limited, and the impact of AFIX on adolescent immunization coverage has not, until present, been rigorously evaluated.

To address this need, we conducted a randomized controlled trial of adolescent AFIX consultations delivered to high-volume primary care clinics in North Carolina. The purpose of this trial was to evaluate the effectiveness of AFIX with regard to raising adolescent immunization coverage and to explore strategies for delivering consultations via interactive webinar versus in-person office visit. In a three-arm trial with 91 clinics, we found that both webinar and in-person AFIX consultations modestly improved immunization coverage among adolescents, ages 11 to 12, compared to no-consultation control (Gilkey, Dayton, Moss, *et al.*, in preparation). At five-month follow-up, clinics in the in-person and webinar conditions outperformed the control condition by three to five percentage points for Tdap and meningococcal vaccine coverage. Clinics in the intervention conditions achieved small, but statistically significant, improvements over the control clinics for HPV vaccination. Overall, our findings suggest that AFIX holds promise for improving adolescent immunization, but that the program needs further development with regard to HPV vaccination before it should be adopted nationally.

Our finding that webinar delivery of AFIX raised adolescent immunization coverage at least as much as in-person delivery warrants further exploration. Webinar delivery could substantially increase the reach of AFIX by eliminating the cost and inconvenience associated with traveling to clinics to deliver in-person consultations. However, in addition to cost, reach is also determined by participants’ willingness to engage in quality improvement efforts. For this reason, webinar delivery must be acceptable to vaccine providers in addition to being low cost. To investigate how delivery mode may impact the reach of adolescent AFIX, we conducted a process evaluation with 61 clinics in this study’s intervention arms to compare in-person and webinar consultations on provider participation and satisfaction as well as program cost.

## Methods

### Participants

We randomly selected 91 of 481 eligible clinics to participate in the study using the North Carolina Immunization Registry (NCIR), the state’s immunization information system. Used by over 90% of vaccine providers in the state, NCIR is a secure, web-based database that contains immunization information for 67% of North Carolina’s adolescents [11]. Eligible clinics were pediatric or family practice clinics with more than 200 patients, ages 11 to 18, with active records in the registry. Each clinic was randomized to one of three conditions: in-person AFIX consultation (30 clinics); webinar AFIX consultation (31 clinics); or no consultation control (30 clinics). This report focuses solely on the 61 clinics assigned to the in-person and webinar intervention conditions; we did not collect process data from the control clinics. The North Carolina Division of Public Health Institutional Review Board approved the study protocol.

### Intervention

From April to August 2011, one immunization specialist (AD) from the North Carolina Division of Public Health delivered one adolescent AFIX consultation to each clinic via interactive webinar or in-person office visit. Prior to each meeting, the specialist prepared for the consultation by using the state’s immunization registry to generate reports related to the clinic’s baseline performance. These reports included: the assessment report, which listed the percentage of the clinic’s adolescent patients, ages 11 to 18, who were up-to-date for targeted adolescent and early childhood vaccines; the missing immunization report, which identified patients who were due for one or more vaccines; and the duplicate client report, which identified patients who had duplicate records in the registry.

The immunization specialist typically delivered the consultation to the nurse who served as the clinic’s designated immunization coordinator, although on several occasions other staff members also attended. Consultations centered on several components:

#### Assessment and feedback

The specialist began by sharing findings of the assessment report that detailed the clinic’s immunization coverage levels. She helped providers contextualize the assessment findings by showing how the clinic compared to others in the county and state.

#### Education and training

The specialist also provided hands-on training in immunization best practices on three topics. First, using the duplicate client report, the specialist demonstrated how providers could clean up registry records by deleting duplicate or inactive records from the clinic’s roster and by updating active records to reflect previously administered, or historical, vaccine doses. Second, using the missing immunization report, the specialist discussed the importance of using reminder/recall systems to contact patients due for vaccines, and she demonstrated how to use the registry’s reminder/recall function. Third, the specialist discussed strategies for reducing missed opportunities for vaccination, which occur when patients receive some, but not all, of the vaccines for which they are due.

#### Goal setting

Lastly, the specialist worked with providers to identify goals for addressing clinic-specific challenges so as to improve the clinic’s adolescent immunization coverage levels. In-person and webinar consultations did not differ in content. Webinar consultations used Adobe Connect (Adobe Systems Incorporated, San Jose, CA), an interactive conferencing platform that supported real-time communication between the specialist and the provider. This software included a screen sharing function that allowed the specialist to remotely control the providers’ computer screen to demonstrate how to manipulate the registry as she would during the course of an in-person office visit. Given the need to orient providers to the software, webinar consultations lasted about 90 minutes on average in contrast to the 60 minutes needed to complete an in-person consultation.

### Procedure

Participants completed four written surveys for each clinic over the course of the study.

#### Baseline survey

Participants completed this 19-item internet survey prior to the AFIX consultation. Closed- and open-ended questions assessed clinic policies and practices related to adolescent immunization.

#### Best practice assessment survey

Participants completed this written survey during the AFIX consultation. Closed-ended questions assessed each clinic’s current level of activity with regard to 20 immunization best practices in four domains: decreasing missed opportunities; developing reminder/recall systems; establishing staff guidelines for immunizations; and implementing data handling procedures (Table 1). Along with the immunization specialist, participants used this survey to select one practice in each domain as a focus for their quality improvement efforts.

**Table 1:** Strategies to improve immunization for 61 clinics receiving AFIX consultations

	**Baseline assessment**^**a**^	**No. of times chosen as improvement goal**
	**Mean (SD)**	
Reducing missed opportunities		
Use all encounters to vaccinate	3.4 (1.0)	24
Use prompts like posters	3.0 (1.4)	13
Standing-orders for vaccination	3.4 (1.6)	11
Use adolescent catch-up schedule	3.9 (1.2)	7
Train staff on contraindications	3.9 (1.1)	6
Developing a reminder/recall system		
Use reminder/recalls	2.2 (1.1)	40
Update patient contact information	3.5 (1.2)	12
Schedule ‘shots only’ visits	3.1 (1.4)	6
Schedule next visits in office	4.3 (0.8)	3
Establishing staff guidelines		
Create immunization teams	3.2 (1.5)	15
Vaccinate when history is in doubt	3.2 (1.4)	14
Encourage well visits for ages 11-12	3.6 (1.4)	13
Provide resources to hesitant parents	3.9 (1.2)	10
Conduct immunization assessments	4.0 (1.3)	7
Administer multiple vaccines if due	4.6 (0.7)	2
Implement data best practices		
Measure clinic’s immunization rates	2.4 (1.3)	30
Update parents’ vaccination records	3.8 (1.2)	14
Flag charts for due/over-due vaccines	3.5 (1.3)	10
Make shot record visible in chart	4.4 (0.9)	4
Require record of historical doses	4.2 (1.1)	3

*Note*. Use of these strategies at baseline was equally common in clinics in the in-person and webinar conditions, except for use of adolescent catch-up schedule (3.2, SD = 1.2 versus 2.5, SD = 1.3) and flag charts for due/over-due vaccines (2.2, SD = 1.4 versus 2.9, SD = 1.2) (both *p* < .05).

^a^Current activity assessed using a five-point response scale ranging from ‘no activity’ (1) to ‘optimal’ (5).

#### Evaluation survey

Participants completed this 12-item internet survey directly after receiving the AFIX consultation. Closed- and open-ended questions assessed participants’ experience of the consultation.

#### Follow-up survey

Participants completed this 21-item internet survey five months after receiving the AFIX consultation. Closed- and open-ended questions assessed participants’ quality improvement activities since the consultation.

### Measures

#### Participation

We assessed how and how much providers chose to engage in quality improvement activities using several sets of measures. First, we assessed baseline organizational practices and goal endorsement using the best practice assessment. Second, we assessed participants’ confidence using reminder/recall systems with one item administered in three surveys: baseline, evaluation, and five-month follow-up. Third, we measured participants’ perceived quality improvement effort via the follow-up survey, which assessed effort related to key programmatic activities, including use of reminder/recall systems.

#### Satisfaction

Satisfaction measures came from the evaluation survey. This questionnaire assessed participants’ perceptions of the AFIX consultation in terms of helpfulness, ease of understanding, convenience, and length of consultation. Participants also rated the importance of individual program components, including: reports on immunization coverage, missing immunizations, and duplicate clients; coverage comparisons at the regional, state, and national levels; and reminder/recall training. Finally, participants indicated whether they would have preferred receiving the intervention via in-person or webinar delivery.

#### Cost

Cost data came from expense tracking logs maintained by the immunization specialist (AD). These logs detailed expenses related to delivering the intervention, including: staff time needed for consultation preparation, delivery and travel; mileage reimbursement and lodging for overnight trips; and mailing and webinar-hosting fees. This analysis did not include costs incurred by participating providers, patients, or parents.

#### Clinic characteristics

The North Carolina Immunization Registry provided data on clinic characteristics, including the proportions of adolescent patients who were black, white, or another race and the proportions of vaccines doses administered at the clinic that were either publicly or privately funded. Publicly-funded doses were those for which a patient was eligible for the Vaccines for Children (VFC) program [12]. VFC is a federally-financed program that provides free vaccines for children and adolescents who are uninsured, Medicaid-insured, or of American Indian or Alaska Native descent; the program also provides vaccines for underinsured youth through federally qualified health centers and rural health clinics. We also assessed clinic specialty (pediatric or family practice) and location. We defined clinics located within a metropolitan statistical area as ‘urban or suburban’ and others as ‘rural’ [13].

### Statistical analysis

To check randomization, we assessed whether study arms differed on clinic characteristics using chi-square tests and analyses of variance. We used *t*-tests to compare clinics in the in-person and webinar conditions on mean scores related to participation (*i.e.*, reminder/recall confidence, quality improvement effort) and satisfaction. We calculated intervention cost per clinic by dividing the total intervention cost for each condition by the number of clinics in that condition. We analyzed data using Stata Version 12.0 (Statacorp, College Station, TX). Statistical tests were two-tailed with a critical alpha of 0.05.

## Results

### Clinic characteristics

Of 61 clinics that received AFIX consultations, about half (56%) specialized in pediatrics, and the rest specialized in family medicine. Clinics served 71,874 adolescent patients with active records in the immunization registry. These records indicated that, on average, clinics’ patients were predominantly white (50%) and black (23%); race was unspecified for one-quarter (25%) of patients in the sample. In each study arm, over one-third (42% on average) of vaccine doses that clinics administered were eligible for public funding through the Vaccines for Children program. Most clinics (77%) were located in urban/suburban versus rural counties. Clinics in the in-person and webinar conditions did not differ on any of the clinic characteristics we assessed.

### Participation

All 61 clinics assigned to the intervention conditions completed full AFIX consultations and responded to the pre-consultation, post-consultation, and five-month follow-up surveys. Overall, 79% of respondents reported sharing the findings of the clinic’s immunization coverage report with others, including one or more of their clinics’ healthcare providers (*n* = 34) or office managers (*n* = 14).

### Strategies for improving immunization rates

Clinics in the in-person and webinar conditions rated their baseline use of immunization best practices similarly. Of 20 strategies for improving immunization rates, participants most often chose to focus their quality improvement efforts on using reminder/recall systems (66%). They also commonly chose measuring the clinic’s immunization rates periodically (49%); using all encounters as opportunities to provide vaccines (39%); and creating immunization teams (25%). These popular strategies were among those that participants rated most poorly in terms of their current activity levels (Table 1).

### Reminder/recalls

**Figure 1:**
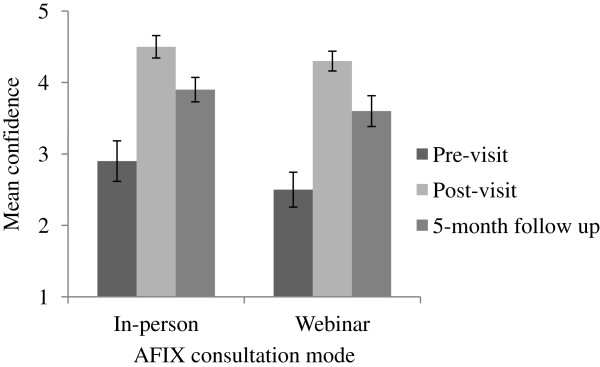
**Confidence in being able to run patient reminder/recalls among staff at clinics in the in-person (*k *= 30) and webinar (*k *= 31) conditions.***Note:* Bars show standard errors.

On a five-point response scale, participants’ mean confidence in running reminder/recalls increased from 2.7 (SD = 1.5) at pre-consultation to 4.4 (SD = 0.8) at post-consultation (*p* < 0.001). Compared to pre-consultation scores, mean confidence remained higher at five-month follow-up (mean = 3.8, SD = 1.1, *p* < 0.001). Participants in the webinar versus in-person conditions reported the same levels of confidence at each of the three time points (Figure 1).

**Figure 2:**
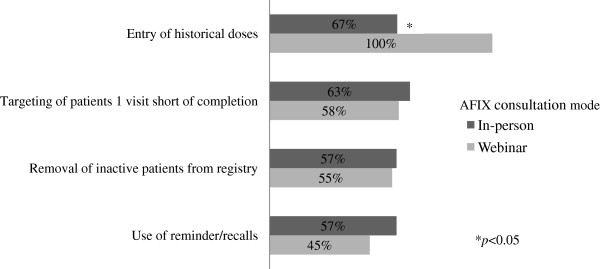
**Clinics reporting more effort in programmatic activities at five-month follow up after in-person (*k =*** **30) and webinar (*k =*** **31) consultations.**

In terms of self-reported use of reminder/recall systems, 51% of respondents reported making ‘more effort’ to use any system between baseline and five-month follow-up, and study arms did not differ on this measure (Figure 2). At follow up, 43% of respondents in the in-person condition and 29% in the webinar condition reported having run at least one recall using the immunization registry. Among these respondents, the average number of recalls completed at follow-up was 1.3 (SD = 1.0) for the in-person condition and 2.6 (SD = 1.8) for the webinar condition. Across intervention conditions, reasons named for not using the registry’s reminder/recall system included lack of staff or time (*n* = 18), the competing challenge of implementing an electronic health record system (*n* = 5), and use of a system other than the registry to perform recalls (*n* = 4).

### Other programmatic activities

At five-month follow-up, 84% of participants reported having made more effort since baseline in entering historical vaccine doses into the registry. Over half of respondents reported making more effort with two other activities: targeting patients one office visit short of being fully immunized (61%) or removing inactive patients from the registry (56%). Of these three activities, we observed statistically significant differences by intervention group for only the entry of historical doses, with more respondents in the webinar versus in-person condition reporting ‘more effort’ (100% vs 67%) (Figure 2).

### Satisfaction

Participants rated AFIX consultations highly in terms of helpfulness (mean = 4.7, SD = 0.5), ease of understanding (mean = 4.7, SD = 0.7), convenience (mean = 4.6, SD = 0.7), and length of consultation (mean = 4.5, SD = 0.7). Participants also gave high marks to the importance of individual program components, including the clinic’s immunization coverage assessment report (mean = 4.6, SD = 0.7), the missing immunization report (mean = 4.7, SD = 0.7), the duplicate client report (mean = 4.3, SD = 0.9), the state and national comparison (mean = 4.2, SD = 0.8), the county and regional comparison (mean = 4.5, SD = 0.8), and the reminder/recall training (mean = 4.5, SD = 0.8). Participants in the two intervention conditions expressed the same high levels of satisfaction on these measures (Table 2). In terms of intervention delivery mode, 30 of 31 participants who received webinar consultations indicated a preference for that mode. All 30 participants who received in-person consultations indicated a preference for in-person delivery.

**Table 2:** Mean satisfaction ratings

	**In-person**	**Webinar**
	**Mean (SD)**	**Mean (SD)**
Overall program satisfaction^a^		
Helpfulness	4.7 (0.6)	4.7 (0.4)
Ease of understanding	4.7 (0.8)	4.7 (0.4)
Convenience	4.5 (0.8)	4.5 (0.8)
Length of consultation	4.7 (0.5)	4.4 (0.8)
Rating of program components^b^		
Assessment report	4.6 (0.6)	4.6 (0.9)
Missing immunization report	4.8 (0.5)	4.6 (0.9)
Duplicate client report	4.4 (0.9)	4.3 (1.0)
State/national comparison^c^	4.2 (0.8)	4.3 (0.9)
County/regional comparison	4.5 (0.8)	4.5 (0.9)
Reminder/recall training	4.4 (0.7)	4.5 (0.9)

*Note.* In-person and webinar conditions had comparable satisfaction scores (all comparisons *p* > 0.05).

^a^Items assessed with a five-point response scale ranging from ‘strongly disagree’ (1) to ‘strongly agree’ (5).

^b^Items assessed with a five-point response scale ranging from ‘very unimportant’ (1) to ‘very important’ (5).

^c^Excludes data missing for clinics in the in-person (n = 1) and webinar (n = 1) conditions.

### Cost

The intervention cost per clinic was $152.45 for the in-person condition and $99.95 for the webinar condition (Table 3).

**Table 3:** Average cost per clinic for AFIX consultations

	**In-person**	**Webinar**
Staffing ($20.51/hour)		
Consultation preparation (2 hours)	$41.02	$41.02
Consultation (1 hour in-person, 1.5 hours webinar)	$20.51	$30.77
Travel to consultation (2 hours)	$41.02	n/a
Travel		
Mileage (125 miles/consultation @ $0.30/mile)	$37.50	n/a
Lodging and meals	$12.40	n/a
Mailings	n/a	$15.58
Webinar license ($390/year)	n/a	$12.58
**Total**	**$152.45**	**$99.95**

*Note*. The 30 in-person AFIX consultations required three overnight trips that incurred lodging and meal expenses of $372.

## Discussion

This process evaluation compared two modes of delivering an immunization quality improvement program to primary care clinics in North Carolina. We found that AFIX consultations delivered by interactive webinar versus in-person office visit elicited similar levels of participation, and healthcare providers in both groups showed sustained improvement with regard to an important intermediate outcome, confidence in using reminder/recall systems. We were also encouraged to find that participant satisfaction was very high overall and comparable between delivery modes. When it came to program cost, however, webinar delivery clearly outperformed the traditional, in-person approach; we found that webinar consultations cost about one-third less per clinic. Taken together, these findings suggest that webinar delivery deserves serious consideration by state health departments seeking to implement adolescent AFIX.

Several factors may explain the somewhat surprising success of webinar delivery. First, the novelty of the videoconferencing software we employed may have elicited interest from our participants, who, as healthcare providers, likely had a higher degree of technological sophistication than the general public. At the very least, we can conclude that any technical difficulties participants faced were not burdensome enough to preclude participation or greatly detract from overall satisfaction. Second, we estimated that, despite covering the same content, webinar consultations lasted about thirty minutes longer on average than in-person consultations; increased interaction time may have supported webinar delivery. Finally, we can report anecdotally that providers often rescheduled webinar consultations, but rarely did so for those delivered in-person. Having greater flexibility may have allowed webinar participants to schedule consultations when they could most fully attend to programmatic activities. Whatever the case, this study adds to a small, but growing body of literature that suggests that internet-based approaches such as videoconferencing are at least as effective as—and perhaps even more effective than—in-person instruction for delivering continuing education to health professionals [14-16].

In terms of programmatic implications, webinar delivery offers three main advantages. First, the lower cost of webinar consultations could dramatically extend the reach of AFIX. In our own study, for example, we could have reached an additional 15 clinics, serving about 17,700 adolescents, had we used the funds spent on in-person consultations for webinar consultations instead. Second, in addition to cost, webinar delivery eliminates the inconvenience and disruption state health department personnel experience when traveling to clinics for in-person consultations. Travel can pose an especially difficult obstacle in states, such as North Carolina, that periodically prohibit state employees from traveling as a way to manage their surprisingly frequent fiscal crises. Third, webinar delivery may also expand who can deliver AFIX by making geographic proximity unnecessary for program implementation. For instance, program officers at CDC could deliver webinar consultations nationwide, thereby centralizing program planning and dissemination and reducing the burden on state health departments [17]. Clinics in rural areas may be especially well-served by webinar visits. The potential advantages of webinar delivery are, thus, substantial in terms of flexibility and efficiency, although these benefits must be balanced against disadvantages such as reduced ability to collect observational data about clinic setting and work flow.

One aspect of our program that warrants special comment is the goal to raise providers’ use of patient reminder/recall systems. Reminder/recall is prioritized in the AFIX program because the strategy is associated with increased immunization rates for both young children and adolescents [18,19]. Providers in our study also prioritized reminder/recall use as a focus of their own quality improvement efforts, and we found that our intervention successfully increased participants’ confidence in using such systems regardless of delivery mode. However, despite goal setting and improved confidence, subsequent use of reminder/recall systems was modest, with only about one-third of participants reporting the activity at five-month follow-up. Lack of staff time was the primary barrier to use. This finding corresponds with existing research, which suggests that conducting reminder/recalls is difficult in the context of primary care because of the burden associated with updating patients’ contact information and then generating calls or mailings [17]. Given the recent success of centralized versus provider-based programs for reminder/recall [17,19], state health departments should carefully consider how much to emphasize reminder/recall training during AFIX consultations since conducting reminder/recalls directly at the state level may ultimately be more effective.

In terms of strengths, this study employed a strong research design, including random allocation of clinics to study conditions, to compare intervention delivery modes among high-volume primary care clinics. Limitations include a modestly-sized, state-specific sample and the use of self-reported measures to assess program participation. Our calculation of program cost per clinic focused narrowly on intervention delivery so as to compare delivery modes; we excluded factors, such as overhead expenses, costs incurred by providers, and quality adjusted life years, that would be needed to determine overall program costs or to conduct a comprehensive cost-effectiveness analysis. One author (AD) delivered all in-person and webinar consultations; this consistency maximized the comparability of the study groups, but other immunization specialists may achieve higher or lower levels of participation and satisfaction. Additional research is needed to understand how our findings generalize to clinics with lower patient volumes, particularly given that small clinics may have reduced access to the technology needed to support webinar delivery.

## Conclusions

Quality improvement programs are urgently needed to address the underuse of adolescent vaccines, and the CDC’s AFIX model offers a strong foundation for these efforts. This study provides evidence to suggest that webinar delivery could substantially increase the reach of AFIX without adversely affecting participant experience. As adolescent AFIX undergoes further development, program planners should continue to pilot consultation materials via webinar. Given that adolescent AFIX will be disseminated nationally, maximizing the program’s efficiency is of vital importance for supporting state health departments as they seek to make the best use of increasingly constrained resources.

## Abbreviations

AFIX: Assessment, Feedback, Incentives, and Exchange; CDC: Centers for Disease Control and Prevention; HPV: Human papillomavirus; NCIR: North Carolina Immunization Registry; Tdap: Tetanus, diphtheria, and pertussis; VFC: Vaccines for Children.

## Competing interests

NB has received HPV vaccine-related grants from or been on advisory boards for GlaxoSmithKline, Merck and Sanofi-Pasteur. The remaining authors (MG, JM, AR, AD, AG) declare that they have no competing interests.

## Authors’ contributions

MG led data analysis and wrote the first draft of the manuscript. JM managed the data and supported the analysis. AR participated in data collection. AD conceived of the study, participated in study design, and led intervention delivery and data collection. AG conceived of the study, participated in study design, and supported data collection. NB conceived of the study, participated in study design, and oversaw data collection, data analysis, and manuscript development. NB confirms that everyone who has made significant contributions to the work is recognized as an author. All authors revised the manuscript critically for important intellectual content and have given final approval of the version to be published.
